
JOURNAL INFORMATION
==============================
NLM Title Abbreviation: Wirtschaftsdienst
Journal ID: Wirtschaftsdienst
NLM Journal ID: 101086612

Wirtschaftsdienst (Hamburg, Germany : 1949)

ISSN: 0043-6275

ARTICLE INFORMATION
==============================
PMCID: PMC8383015
PMID: 34456387
DOI: 10.1007/s10273-021-2987-1
Article ID: 2987
Article version: 1
Subjects: Zeitgespräch

Der Vorteil des Experimentierens in der Pandemie

Konrad Kai A. Kai.Konrad@tax.mpg.de
1
Thum Marcel marcel.thum@tu-dresden.de
2
1 grid.461808.3 0000 0004 0584 1597 Öffentliche Finanzen, Max-Planck-Institut für Steuerrecht, Marstallplatz 1, 80539 München, Deutschland
2 grid.4488.0 0000 0001 2111 7257 Fakultät Wirtschaftswissenschaften, Technische Universität Dresden, Helmholtzstr. 10, 01069 Dresden, Deutschland
Electronic publication date: 2021 Aug 24
Print publication date: 2021
Volume: 101
Issue: 8
First page: 603
Last page: 605
Copyright: © Der/die Autor:in(nen) 2021
License: Open Access: Dieser Artikel wird unter der Creative Commons Namensnennung 4.0 International Lizenz veröffentlicht (https://creativecommons.org/licenses/by/4.0/deed.de).
Open Access wird durch die ZBW — Leibniz-Informationszentrum Wirtschaft gefördert.
License URL: https://creativecommons.org/licenses/by/4.0/

issue-copyright-statement: © The Author(s) 2021

==============================

Während der Corona-Pandemie setzte die schwedische Regierung konsequent auf die Eigenverantwortung der Bürger:innen und ließ das öffentliche Leben weitgehend unreguliert weiterlaufen. Dafür wurde die schwedische Regierung in den internationalen Medien und in der europäischen Politik zum Teil heftig kritisiert. Für einen schwedischen Bürger, der um seine Gesundheit besorgt ist, mag die Kritik einer zu großen Risikoübernahme berechtigt sein. Warum jedoch aus anderen Ländern so viel Kritik auf die schwedische Politik einprasselt, ist weniger gut verständlich. Denn schließlich hat der schwedische Weg dem Rest der Welt ein Experimentallabor geliefert. Die anderen Länder haben doch davon profitiert, dass

Schweden Informationen zur Wirksamkeit einer weitgehenden Laissez-faire-Politik produziert hat. Wenn das schwedische Modell scheitert, muss man die Politik in anderen Ländern nicht nachahmen. Wäre der schwedische Sonderweg hingegen ein voller Erfolg, wäre dies eine wichtige und nützliche Information, an der man sich in späteren Phasen der Pandemie oder bei künftigen Pandemien ausrichten könnte.

Wir entwickeln in diesem Beitrag ein Argument dafür, dass aus globaler Sicht die Krisenreaktionen tendenziell zu homogen waren: Abweichende Politiken liefern wichtige Erkenntnisse. Angesichts von positiven Erfahrungsexternalitäten wird aus globaler Sicht zu wenig experimentiert. Durch eine internationale Koordination, wer in welchen Regionen welche alternativen Politikmaßnahmen austestet, hätte die Politik wichtige Erkenntnisse für die Bekämpfung dieser und möglicherweise kommender Pandemien gewinnen können.

## Politisches Experimentieren

Regionen können aus Fehlern und Erfolgen anderer Regionen lernen. Allgemein wurde der Sachverhalt von Cai und Treisman (2009) im Kontext föderaler Staaten herausgearbeitet. Eine einzelne Region, die sich für eine Politikmaßnahme entscheidet, von der man nicht weiß, ob sie nützt oder schadet, genießt die Vorteile im Erfolgsfall und spürt die Nachteile der Maßnahme im Misserfolgsfall. Treten Erfolg und Misserfolg beispielsweise mit gleicher Wahrscheinlichkeit ein, beträgt der Gewinn im Erfolgsfall aber 100 und der Verlust im Misserfolgsfall -200, zahlt sich die Maßnahme im Erwartungswert für die Region nicht aus und wird deshalb typischerweise nicht ergriffen. Trotzdem wäre es gut, wenn eine der Regionen das Experiment wagen würde: Schadet die Maßnahme, muss man sie andernorts ja nicht nachahmen. Ist sie erfolgreich, könnte sie von anderen Regionen (oder Ländern) ebenfalls angewendet werden. Das Experimentieren einer Region (eines Landes) hat im interregionalen (internationalen) Kontext der Krise also eine positive Externalität.

Anreize von Politiker:innen können die genannten Gründe für ein zu geringes Ausmaß an Experimentieren und Lernen verstärken und ergänzen. Die Literatur beschreibt die Reputationsüberlegungen von Manager:innen, die unter Erfolgsdruck stehen, und wie dies zu Herdenverhalten führen kann (Zwiebel, 1995). Reputationsüberlegungen von unter Erfolgsdruck stehenden Politiker:innen können aus ganz analogen Gründen zu Herdenverhalten führen und damit zu einer zu großen Homogenität in den Krisenreaktionen. Die Arbeiten zur Theorie der Electoral Accountability (beispielsweise die Übersichtsarbeit von Ashworth, 2012) geben Anhaltspunkte für die dafür ursächlichen Mechanismen.[1] Politische Entscheidungen und ihre Konsequenzen werden von den Wählenden als informative Signale interpretiert. Sie versuchen, aus den Entscheidungen und ihren tatsächlichen späteren Auswirkungen, Informationen über die ideologische Position und vor allem über die Kompetenz der amtierenden Politiker:innen zu gewinnen. Canes-Wrone et al. (2001) beispielsweise untersuchen, wann ein Politiker oder eine Politikerin mit privater Information über die Wahrscheinlichkeitsverteilung der tatsächlichen Vorteilhaftigkeit von Politikalternativen entsprechend diesem Wissen entscheidet oder einfach das tut, was die Wähler:innen angesichts ihrer schlechteren Informationssituation ex ante für richtig halten. Hierzu ein Beispiel: Ein Politiker, der in einer bestimmten schwierigen Entscheidungsfrage mit unsicherem Ausgang so handelt wie alle seine Kolleg:innen in anderen Ländern, geht damit auf Nummer sicher. Ob sich die Entscheidung im Nachhinein als richtig oder falsch erweist, verrät wenig über seine eigenen Fähigkeiten. Ein Politiker, der anders als alle seine Kolleg:innen entscheidet, wird im Nachgang je nach Ergebnis blamiert dastehen oder als mutige, weise Staatsperson erscheinen.

Wie beispielsweise die Arbeit von Effinger und Polborn (2001) zeigt, könnten Reputationsüberlegungen in sequenziellen Entscheidungssituationen grundsätzlich auch zu Anti-Herding führen. Wichtig für die Entscheidung für oder gegen Herdenverhalten ist z. B. die Frage, ob der Politiker nur einfach Negativsignale vermeiden will

1 Politökonomische Logik wurde ebenfalls auf Fragen der Pandemiebekämpfung angewendet. Eine Übersicht über einige wichtige Aspekte geben Boettke und Powell (2021). Kaplan et al. (2021) betonen die unterschiedliche Betroffenheit verschiedener Bevölkerungsgruppen, z. B. jung und alt oder reich und arm. Coyne et al. (2021) betonen, dass die politische Antwort auf eine Pandemie nicht die Entscheidung eines wohlmeinenden Zentralplaners ist, sondern das Handlungsergebnis einer Vielzahl von Akteur:innen und deren Einzelentscheidungen. Beispiele sind die Konflikte zwischen unterschiedlichen Regierungsebenen und die Rent-seeking-Problematik, die sich in einer Pandemie eröffnet.

(was üblicherweise für die Wiederwahl ausreicht) oder an einem Wettbewerb teilnimmt, der weit überdurchschnittliche Kompetenz belohnt.

Wenn es für den weiteren beruflichen Erfolg in der Politik ausreicht, nicht schlechter als andere zu erscheinen, dann tendiert die Person dazu, die sichere Option zu wählen. Solche Überlegungen mögen ein vergleichsweise homogenes internationales Entscheidungsverhalten der Politik in der Pandemie erklären.

Die Frage, ob die Politik in der Pandemie Herdenverhalten zeigt, ist wohlfahrtstheoretisch relevant: Auch wenn Herdenverhalten für die Entscheidungstragenden individuell rational sein mag, verhindert es ein optimales Maß an Experimentieren und Lernen. Mögliche Wohlfahrtsgewinne aus einer stetigen Verbesserung der Krisenreaktion bleiben ungenutzt. Für das Entscheidungsverhalten der Politik spielt wohl auch die mediale Verarbeitung von Nachrichten über Konsequenzen von Politikentscheidungen eine Rolle.

In einer Medienwelt, in der Fehlentscheidungen besonders breit angeprangert werden, mag das die Politik zu besonderer Vorsicht mahnen. Die mediale Verbreitung der Bilder von den Pandemieopfern in Bergamo könnte z.B. dafür gesorgt haben, dass Politiker:innen die Reserven mit Intensivbetten stark ins Zentrum der Aufmerksamkeit gerückt haben.

### Folgerungen für die Pandemiepolitik

Überträgt man die obigen Überlegungen auf die Pandemiepolitik, deutet die positive Informationsexternalität für sich genommen auf ein zu geringes Niveau von Experimentierfreudigkeit in der internationalen Pandemiebekämpfung hin. Ohne internationale Koordination, wer welche Politikmaßnahme systematisch testet, werden zu wenige unterschiedliche Gegenmaßnahmen ausprobiert.

Man könnte vielleicht hoffen, dass ein gewisses Maß an natürlichen Experimenten alleine aus der Heterogenität der politischen Systeme, der institutionellen Rahmenbedingungen und Wählerpräferenzen resultiert. Chiplunkar und Das (2021) berichten über die Wirkung von Unterschieden im politischen System und der politischen Führung auf die Wahl der Krisenreaktion. Tendenziell erließen autoritärere Regierungen auch strengere Maßnahmen, um die Mobilität der Bevölkerung zu reduzieren (Frey et al., 2020). Auch die Reaktionsgeschwindigkeit in der Pandemiepolitik unterscheidet sich zum Teil erheblich (Förtsch und Knoll, 2020). Das mag zu Heterogenität in den Krisenreaktionen führen, vergleichbar mit einem Experimentieren. Ein perfektes Experiment im Sinne kausalanalytischer Studien liegt damit natürlich nicht vor. Denn eine Bevölkerung, die sich eine autoritärere Regierung wählt, verhält sich vielleicht auch in vielen weiteren Dimensionen anders als eine eher liberale Gesellschaft.

Daher ist zu vermuten, dass das Gewinnpotenzial, das im Experimentieren liegt, am besten durch internationale Koordination auf lokal heterogene Krisenreaktionen ausgeschöpft werden kann. Ähnliche Länder könnten sich darauf einigen, in gezielt ausgewählten Regionen abweichende Politiken zu testen und die Erfahrungen daraus systematisch auszuwerten.

Die Informationsexternalitäten stehen natürlich neben einer langen Liste von anderen externen Effekten, die es in einer Pandemie zwischen Regionen oder Ländern gibt. Zu nennen sind die medizinischen und wirtschaftlichen Interdependenzen und die Mobilität von Personen zwischen Regionen. Wie stets entscheidet sich die Frage des richtigen „wie" oder des „zu viel oder zu wenig" aus der Abwägung einer Vielzahl von Effekten. Bemerkenswert ist dabei, dass die Interdependenzen zwischen Regionen auch das Experimentieren erschweren. So wurde im föderalen Kontext in Deutschland nicht ganz zu Unrecht darauf verwiesen, dass Alleingänge einzelner Regionen unmittelbar auf die Pandemie- und Wirtschaftsentwicklung der Nachbarregionen wirken. Dieses Problem ist auf Gemeindeebene sicher am größten, auf der Ebene von Bundesländern schon geringer und am geringsten, wenn man Entwicklungen unterschiedlicher Politiken auf der Ebene von Nationalstaaten vergleicht.

Prof. Dr. Kai A. Konrad ist Direktor am Max-Planck-Institut für Steuerrecht und Öffentliche Finanzen in München.

Prof. Dr. Marcel Thum lehrt Volkswirtschaftslehre, insbesondere Finanzwissenschaft, an der Technischen Universität Dresden und ist Direktor des ifo Dresden.


Literatur

Ashworth S Electoral Accountability: recent theoretical and empirical work Annual Review of Political Science 2012 15 183 201 10.1146/annurev-polisci-031710-103823
Boettke P Powell B The political economy of the COVID-19 pandemic Southern Economic Journal 2021 87 4 1090 1106 10.1002/soej.12488 PMC8014844 33821055
Cai H B Treisman D Political decentralization and policy experimentation Quarterly Journal of Political Science 2009 4 1 35 58 10.1561/100.00008039
Canes-Wrone B Herron M C Shotts K W Leadership and pandering: A theory of executive policymaking American Journal of Political Science 2001 45 3 532 550 10.2307/2669237
Chiplunkar G Das S Political institutions and policy responses during a crisis Journal of Economic Behavior & Organization 2021 185 647 670 10.1016/j.jebo.2021.03.018 PMC9754700 36540422
Coyne C J Duncan T K Hall A R The political economy of state responses to infectious disease Southern Economic Journal 2021 87 4 1119 1137 10.1002/soej.12490 PMC8014786 33821053
Effinger M R Polborn M R Herding and anti-herding: A model of reputational differentiation European Economic Review 2001 45 3 385 403 10.1016/S0014-2921(00)00070-2
Förtsch M Knoll S Wer macht den Anfang? — Regierungsformen und die Reaktionen auf COVID-19 ifo dresden berichtet 2020 3 3 6
Frey C B Chen C Presidente G Democracy, culture, and contagion: Political regimes and countries’ responsiveness to Covid-19 Covid Economics 2020 18 222 238
Kaplan, S., J. Lefler und D. Zilberman (2021), The political economy of COVID-19, Applied Economic Perspectives and Policy.10.1002/aepp.13164 PMC8250203 34230850
Zwiebel J Corporate conservatism and relative compensation Journal of Political Economy 1995 103 1 1 25 10.1086/261973
